# CRISPR/Cas9-mediated genome editing induces exon skipping by alternative splicing or exon deletion

**DOI:** 10.1186/s13059-017-1237-8

**Published:** 2017-06-14

**Authors:** Haiwei Mou, Jordan L. Smith, Lingtao Peng, Hao Yin, Jill Moore, Xiao-Ou Zhang, Chun-Qing Song, Ankur Sheel, Qiongqiong Wu, Deniz M. Ozata, Yingxiang Li, Daniel G. Anderson, Charles P. Emerson, Erik J. Sontheimer, Melissa J. Moore, Zhiping Weng, Wen Xue

**Affiliations:** 1 RNA Therapeutics Institute, University of Massachusetts Medical School, Worcester, MA 01605 USA; 2 David H. Koch Institute for Integrative Cancer Research, Massachusetts Institute of Technology, Cambridge, MA 02142 USA; 3 Program in Bioinformatics and Integrative Biology, University of Massachusetts Medical School, Worcester, MA 01605 USA; 4 Department of Bioinformatics, School of Life Science and Technology, Tongji University, Shanghai, People’s Republic of China; 5 Department of Chemical Engineering, Massachusetts Institute of Technology, Cambridge, MA 02142 USA; 6 Harvard-MIT Division of Health Sciences & Technology, Cambridge, MA 02139 USA; 7 Institute for Medical Engineering and Science, Massachusetts Institute of Technology, Cambridge, MA USA; 8 Wellstone Muscular Dystrophy Program, Department of Neurology, University of Massachusetts Medical School, Worcester, MA 01605 USA; 9 Program in Molecular Medicine, University of Massachusetts Medical School, Worcester, MA 01605 USA; 10 Department of Biochemistry and Molecular Pharmacology, Howard Hughes Medical Institute, University of Massachusetts Medical School, Worcester, MA 01605 USA; 11 Department of Molecular, Cell and Cancer Biology, University of Massachusetts Medical School, 368 Plantation Street, Worcester, MA 01605 USA

## Abstract

CRISPR is widely used to disrupt gene function by inducing small insertions and deletions. Here, we show that some single-guide RNAs (sgRNAs) can induce exon skipping or large genomic deletions that delete exons. For example, CRISPR-mediated editing of β-catenin exon 3, which encodes an autoinhibitory domain, induces partial skipping of the in-frame exon and nuclear accumulation of β-catenin. A single sgRNA can induce small insertions or deletions that partially alter splicing or unexpected larger deletions that remove exons. Exon skipping adds to the unexpected outcomes that must be accounted for, and perhaps taken advantage of, in CRISPR experiments.

**Electronic supplementary material:**

The online version of this article (doi:10.1186/s13059-017-1237-8) contains supplementary material, which is available to authorized users.

## Background

CRISPR/Cas9 genome editing has transformed the study of gene function in many organisms [1–5]. Guide RNAs direct the Cas9 nuclease to create double-strand DNA breaks at complementary target sites in the genome. Repair of these double-strand DNA breaks by non-homologous end-joining (NHEJ) often introduces small insertions or deletions (indels) that shift the open reading frame, thereby inactivating the target gene. CRISPR therefore provides a simple way to generate loss-of-function (LOF) mutations in virtually any gene in the mammalian genome [1]. Nonetheless, CRISPR can also induce off-target editing at genomic positions that imperfectly match the single-guide RNA (sgRNA) sequence, which calls for the implementation of strategies to reduce off-target effects [6, 7]. Besides off-target editing, it remains unknown whether CRISPR-mediated editing has unintended consequence at the post-transcriptional level of the target gene.

We have previously used in vivo delivery of CRISPR to inactivate tumor suppressor genes in mice [8–10]. We also showed that CRISPR can edit oncogenes or disease genes through homolog-directed repair [8, 11, 12]. Here we show that CRISPR-mediated editing of mammalian exons can induce exon skipping. Exon skipping can result from alternative splicing or from genomic deletions that remove exons. Moreover, exon skipping can produce messenger RNAs (mRNAs) with intact reading frames that encode functional proteins.

## Results

**Fig. 1:**
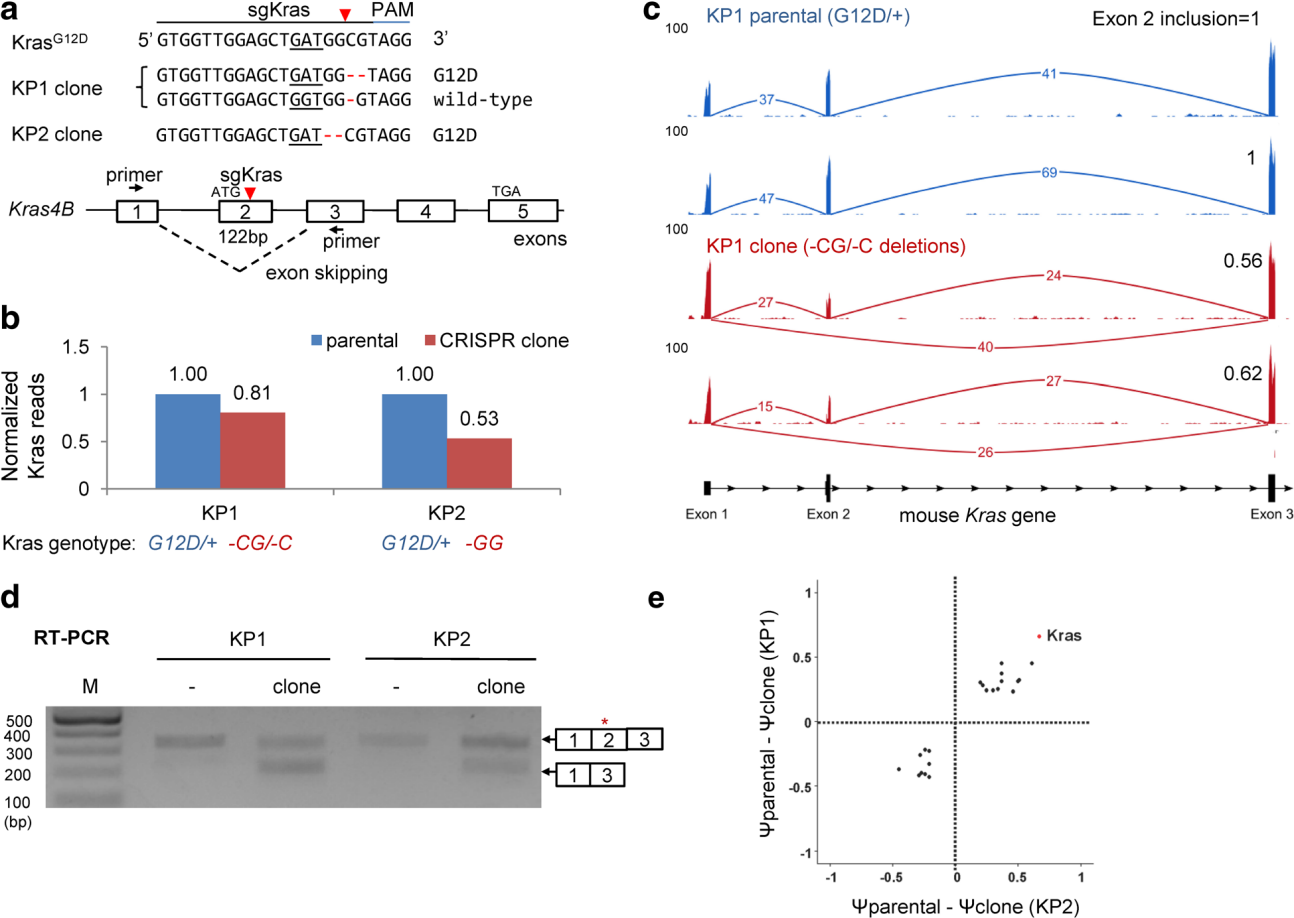
sgRNA targeting Kras induces exon skipping in single cell clones. **a** *Schematic* of an sgRNA targeting exon 2 of the mouse *Kras* gene (sgKras). The *red arrowhead* denotes the Cas9 cleavage site. KP1 and KP2 cell lines were transduced with lentivirus that encodes Cas9 and sgKras. Two single-cell clones (KP1 clone and KP2 clone) harbor frameshift deletions. *Black arrows* indicate the positions of reverse transcription polymerase chain reaction (RT-PCR) primers. The G12D codon is *underlined*. **b** Normalized *Kras* read counts from RNA-sequencing (RNA-seq) analysis of KP parental cells (*blue*) and KP clones (*red*). RNA-seq was done twice for KP2 clone and three times for the other groups. “+” denotes WT allele. **c** RNA-seq showing partial exon 2 skipping in KP1 clones. RNA-seq numbers indicate reads spanning the indicated exon junctions. Two representative biological replicates are shown. **d** RT-PCR analysis of *Kras* mRNA detects an exon 2 skipped band. The expected band sizes are 331 bp and 209 bp. M, molecular marker. “*” denotes indels in PCR products from clones. **e** *Scatter plot* showing 22 exon events that change in both KP1 and KP2 clones. Exclusion of Kras exon 2 is the most frequent event. Ψ, Percentage Splicing Index

We recently used CRISPR to disrupt the *Kras* oncogene in two independent lung adenocarcinoma cell lines [13], which were derived from *Kras* ^*G12D*^; *p53* ^*fl*/*fl*^ (KP) mice [14, 15]. We isolated two single-cell clones each carrying frameshifting deletions in exon 2 (Fig. 1a and Additional file 1: Figure S1a): KP1 carries a 2-nt “-CG” deletion in the G12D allele and a 1-nt “-C” deletion in the otherwise wild-type (WT) *Kras* allele; and KP2 carries a 2-nt “-GG” deletion. Neither clone produces full length Kras protein [13], indicating that all three deletions disrupt the *Kras* reading frame.

Frameshift mutations in early exons are known to trigger nonsense-mediated decay (NMD) [16], which eliminates mRNAs with premature termination codons. When we analyzed mRNA-sequencing (RNA-seq) data, however, we found that apparent *Kras* mRNA levels (i.e. total normalized mRNA reads) were only reduced by 19% in KP1 cells and 47% in KP2 cells, compared with parental KP cells (Fig. 1b). Both clones produced fewer exon 2 reads, but normal levels of exon 1 and 3 reads (Fig. 1c), suggesting that exon 2 might be skipped in the KP1 and KP2 clones. Indeed, we detected exon 1-3 junction reads, indicating that exon 2 was skipped (Fig. 1c and Additional file 1: Figure S1b). Calculating the ratio between exon 2 reads and total reads, we found that exon 2 is included in only 64.0 ± 9.1% of *Kras* reads from KP1-clone (Fig. 1c and Table 1). Similar exon 2 skipping was observed in KP2-clones (Additional file 1: Figure S1c). Concordantly, reverse transcription of *Kras* mRNA followed by polymerase chain reaction (RT-PCR) yielded two products: one corresponded to intact *Kras* complementary DNA (cDNA) and the other corresponded to the exon 1-3 isoform (Fig. 1d). The exon 1-3 isoform retains a partial Kras open reading frame that could initiate translation from an ATG codon in exon 3 (Additional file 1: Figure S2) and produce a severely truncated Kras protein.

**Table 1:** Genomic lesion and mRNA splicing results of single cell clones

Gene/clone	sgRNA target	Allele	Genomic lesion	Exon inclusion (%)
Kras (KP1)	Exon 2	1	-CG	64.0 ± 9.1^b^
		2	-C	
Kras^a^ (KP2)	Exon 2	1	-GG	68.0 ± 7.1^b^
Ctnnb1^c^	Exon 3	1	-CCA	100
		2	832 bp deletion	New mRNA isform with part of intron 2 and exon 4
p65 clone 15	Exon 6	1	+A	100
		2	2.2 kb deletion (exons 5, 6, 7)	ND
p65 clone 31^a^	Exon 6	1	+A	100

^a^Clones with one allele

^b^% exon inclusion is mean ± s.d. (n = 3 for KP1 and n = 2 for KP2)

^c^Clone in Additional file 1: Figure S6

*ND* not determined

Editing of *Kras* did not induce alternative splicing genome-wide. We identified 97 alternatively spliced exons in KP1 cells and 177 events in KP2 cells. KP1 and KP2 clones shared 22 cassette inclusion or exclusion events, with the exclusion of *Kras* exon 2 being the greatest change in both clones (Fig. 1e and Additional file 1: Table S3). Thus, editing of *Kras* exon 2 specifically induced skipping of *Kras* exon 2. Notably, whereas mouse *Kras* ^*G12D*^ (GGU to GAU) transcripts do not skip exon 2 in parental KP cells, we found that ~15% of human *KRAS* ^*G12S*^ (codon 12 GGU to AGU) transcripts skip exon 2 in the A549 human lung cancer cell line (Additional file 1: Figure S1d). We were unable to predict the gain or loss of exon splice enhancers or silencers [17], but our data suggest that sequences near *Kras* codon 12 promote exon 2 inclusion in mouse and human *Kras*. Exon skipping induced by CRISPR editing was not limited to Kras or to mouse KP cells. A recent study showed that CRISPR editing of *FLOT1* exon 3 in HeLa cells can cause skipping of exon 3, exon 4, or exons 3, 4, and 5 [18]. We also detected infrequent exon skipping when we targeted exon 11 of *LMNA* in human HCT116 cells (Additional file 1: Figure S3). Skipping *LMNA* exon 11 produces an in-frame transcript that could be translated into a neomorphic protein.

**Fig. 2:**
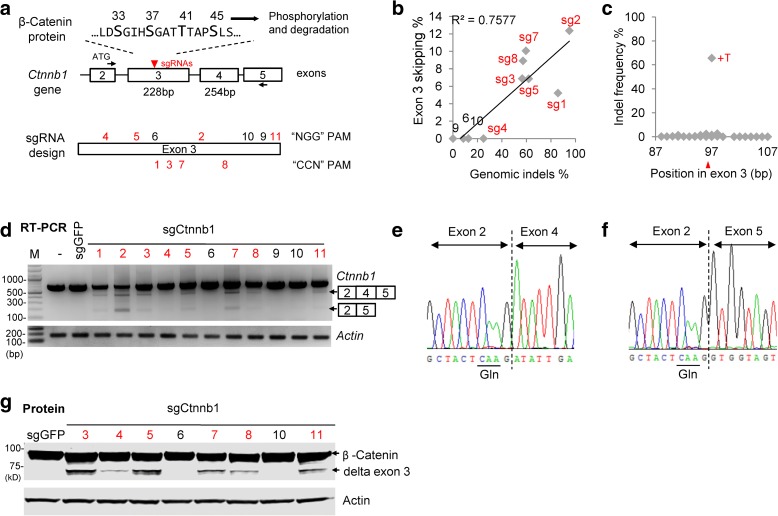
*Ctnnb1* sgRNAs targeting exon 3 induces exon skipping. **a** *Schematic* of the *Ctnnb1* gene. The in-frame exon 3 encodes an inhibitory domain: phosphorylation amino acids 33, 37, 41, and 45 promotes degradation of the β-Catenin protein. Loss of exon 3 stabilizes β-Catenin. Eleven sgRNAs were designed to target exon 3: strong sgRNAs in *red* and weak sgRNAs in *black*, respectively. sgRNAs that use “NGG” PAM are shown above exon 3 and those that use “CCN” PAM are shown below exon 3. **b** Correlation between exon 3 skipping and sgRNA efficiency. Genomic indels were measured by deep sequencing. KP cells were infected with lentivirus. Exon 3 skipping efficiencies are from (**d**). Indels of sg11 were not determined. sgRNAs that induce > 20% indels are marked in *red*. **c** Distribution of sg1 indels shows that a T insertion (+T) at the Cas9 cleavage site nucleotide 97 of exon 3 (*red arrowhead*) was the most frequent. PAM sequence is in *blue*. **d** RT-PCR using primers spanning exons 2 and 5 shows partial exon skipping. *M* molecular marker. sgGFP is a control sgRNA. Exon 3 skipping bands were quantified using ImageQuant TL software and normalized to full length cDNA bands. sg4 showed visible weak bands that could not be quantified. **e**, **f** TOPO cloning and Sanger sequencing confirmed that the two major lower RT-PCR bands in (**c**) are alternative splicing of exon 2-4 and exon 2-5, respectively. **g** Western blot analysis of β-Catenin. Full length β-Catenin is ~86 kD. β-Catenin without exon 3 (delta exon 3) is ~77 kDa. Actin served as a loading control

**Fig. 3:**
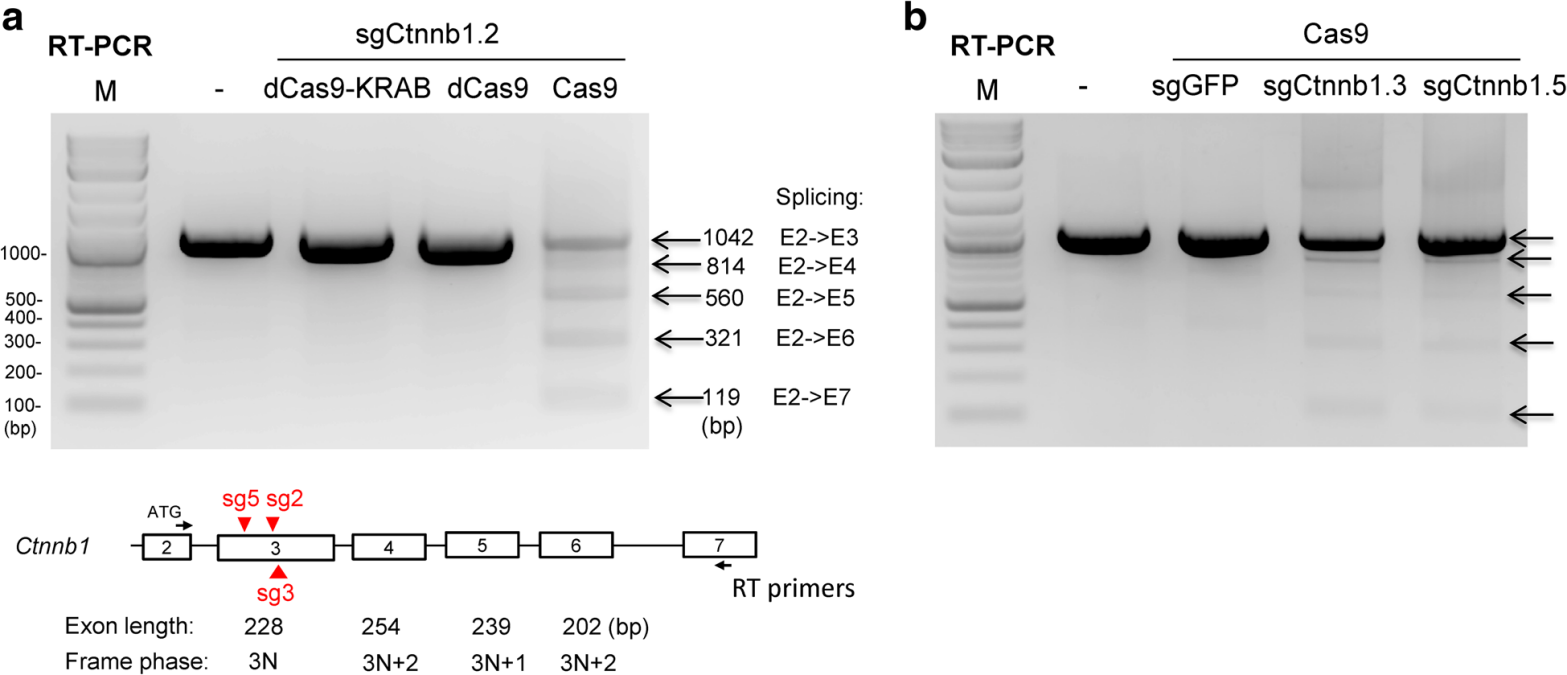
Cas9 nuclease activity required for skipping of one or more exons. **a** RT-PCR analysis of *Ctnnb1* mRNA in KP cells transduced with lentiviruses that encode sgCtnnb1.2 and nuclease-defective Cas9 (dCas9), dCas9-KRAB fusion, or WT Cas9. RT-PCR was performed using primers in exons 2 and 7 on transduced KP cell populations after puromycin selection and FACS sorting. The exon length and reading frame phase are shown. Only the exon 2-4 splice product retains an in-frame β-Catenin coding sequence. **b** RT-PCR analysis of *Ctnnb1* mRNA in KP cells transduced with lentiviruses that encode Cas9 and sgGFP, sg3, or sg5. “–”, untreated

To further explore the idea that exon skipping could produce a functional in-frame transcript, we asked whether CRISPR-mediated editing of *Ctnnb1* exon 3 might induce exon skipping and cause a gain-of-function phenotype. Exon 3 of *Ctnnb1* encodes phosphoacceptor residues that promote degradation of the β-Catenin transcription factor [19]; genetic excision of *Ctnnb1* exon 3—which is in frame with exon 4—stabilizes a constitutively active β-Catenin that accumulates in the nucleus [20, 21]. We designed 11 sgRNAs that target regions along *Ctnnb1* exon 3 (*Ctnnb1*-sg1 to -sg11), transduced individual sgRNAs into KP cells, and used high-throughput sequencing to analyze the extent of editing at the sgRNA target site in each line (Fig. 2b x-axis, Additional file 1: Figure S4 and Additional file 2: Table S4). Three sgRNAs (sg6, sg9, and sg10) inefficiently targeted *Ctnnb1*. Eight of the *Ctnnb1* sgRNAs (sg1 to sg5, sg7, sg8, and sg11), however, induced indels at their target sites with frequencies that exceeded 20%. For example, *Ctnnb1*-sg1 generated + T insertions in about 65% of reads (Fig. 2c). In each population targeted by a strong *Ctnnb1* sgRNA, we detected three RT-PCR products that span exons 2 to 5 (Fig. 2d). The major product corresponds to the normally spliced transcript that includes exon 3. The other two products correspond to alternatively spliced transcripts: one that skips exon 3 (i.e. exon 2-4 splicing, Fig. 2e) and one that skips both exons 3 and 4 (i.e., exon 2-5 splicing, Fig. 2f). *Ctnnb1* sgRNAs targeting either DNA strand induced exon skipping and Cas9 nuclease activity was essential for exon skipping (Fig. 3a).

Western blot analysis revealed that cell populations transduced with the strong sgRNAs produce a smaller ~74 kD β-Catenin protein that corresponds in size to that expected from the exon 2-4 splice product (Fig. 2g). The full length β-Catenin protein was not significantly depleted four days after transduction. To test whether the alternative splicing is dependent on the continuous expression of Cas9 or sgRNA in the lentiviral vectors, we co-transfected Cas9 and *Ctnnb1*-sg1 or a non-targeting sgRNA control. Seven days after transfection, when transfected Cas9 and guide RNAs should be depleted, we examined β-Catenin localization by immunofluorescence. In mouse fibroblast cells transfected with a non-targeting control sgRNA, β-Catenin localized to cell junctions (Additional file 1: Figure S5a). By contrast, in many cells transfected with *Ctnnb1*-sg1, we detected β-Catenin in the nucleus (Additional file 1: Figure S5a). These results suggest that continuous editing is not required for exon skipping and that exon 3 skipping induced by CRISPR-mediated editing of *Ctnnb1* exon 3 produces a gain-of-function β-Catenin isoform.

We further analyzed transcripts spanning exons 2 to 7 in cell populations treated with *Ctnnb1*-sg2, -sg3, and -sg5. In addition to the full-length isoform, we detected four transcripts with exon 2 apparently spliced to each downstream exon (i.e. exon 2-4, exon 2-5, exon 2-6, and exon 2-7; Fig. 3a, b). We do not understand the mechanism of this apparently promiscuous exon skipping induced by *Ctnnb1* exon 3 editing, nor have we been able to correlate promiscuous exon skipping with specific target sites or indel mutations in exon 3. Nevertheless, we isolated a *Ctnnb1*-sg3 edited clone that suggests a potential mechanism (Additional file 1: Figure S6a). This biallelic clone contains a 3-bp in-frame deletion on one allele and a large 832-bp deletion on the other; the 832-bp deletion fuses the 5’ end of intron 2 to the 3’ end of exon 4 (Additional file 1: Figure S6). We detected two transcripts in these cells: the properly spliced transcript that includes the 3-bp deletion and a transcript that includes intron 2 fused to exon 4 (Additional file 1: Figure S6c and Table 1). These results suggest that apparent exon skipping detected in populations of edited cells could reflect genome rearrangements that remove exons.

Two experiments support the idea that a single sgRNA can induce large genomic deletions that remove exons. For example, we isolated 15 clones from mouse 3T3 cells transiently transfected with Cas9 and *Ctnnb1*-sg1, and found that four clones (i.e. clones 4, 5, 13, and 15) showed apparent exon skipping by RT-PCR. Genomic PCR revealed genome rearrangements in three of these clones: large deletions (>500 bp) and smaller deletions (~100 bp) in clones 4 and 15, and large insertions in clones 13 and 15 (Additional file 1: Figure S7). Moreover, after targeting exon 6 of *p65*/*RelA*, we isolated a biallelic *p65* clone (#15): one allele harbors a 1-nt “+A” insertion and the other harbors a 2268-bp deletion that removes exons 5, 6, and 7 (Additional file 1: Figure S8a, c–e). In p65 clone #15, we detected the fully spliced transcript and an exon 4-8 splice product (Additional file 1: Figure S8c). Both alleles encode frameshifted transcripts and both p65 transcripts are present at lower levels than WT (Additional file 1: Figure S8b). We also isolated an edited *p65* clone (#31) homozygous for the same + A insertion as in clone #15, but clone #31 does not produce alternatively spliced transcripts. Thus, the exon 4-8 spliced transcript in clone #15 results from the deletion of exons 5, 6, and 7. These large exon deletion events were unexpected and would be missed using typical PCR-based screening assays.

**Fig. 4:**
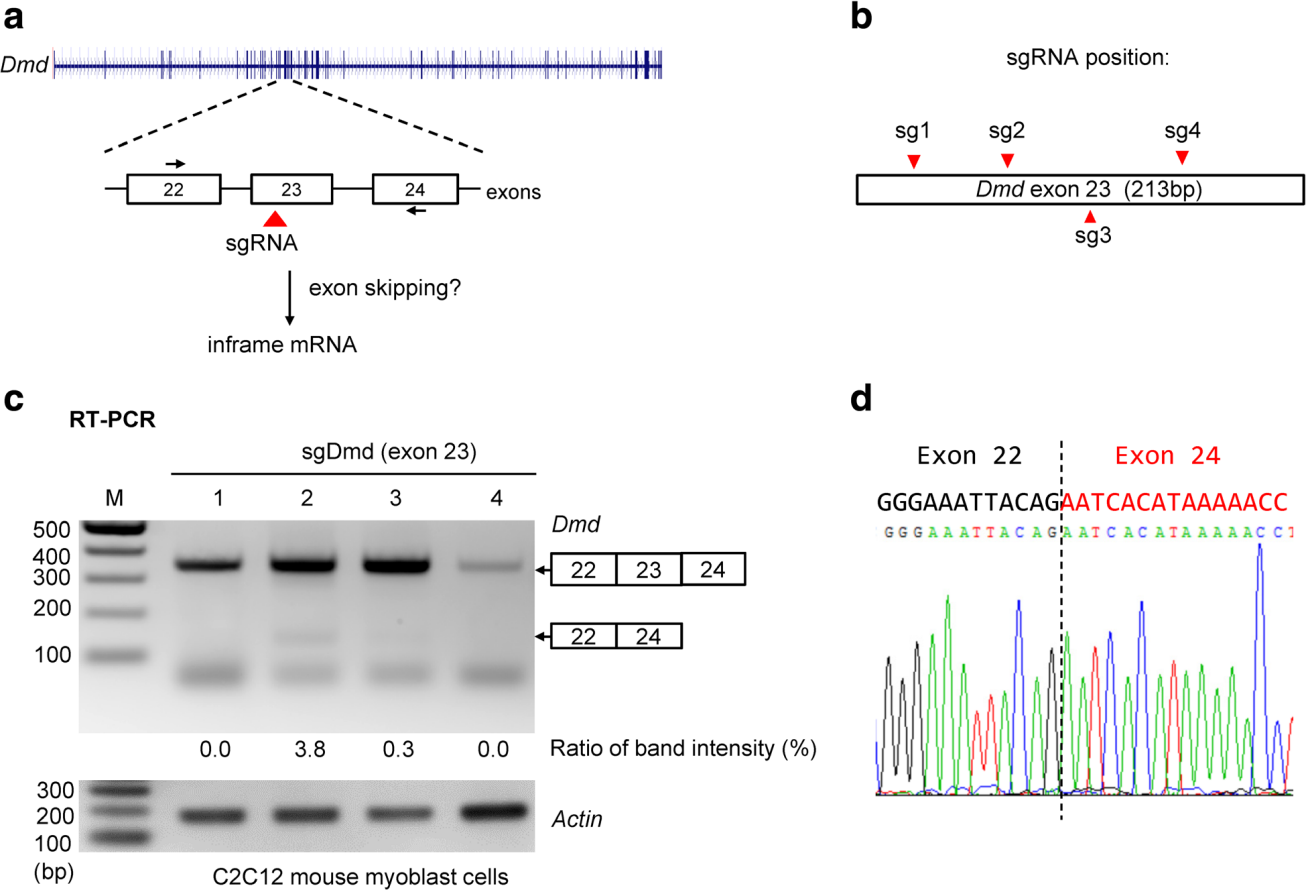
An sgRNA targeting exon 23 of *Dmd* can partially restore in-frame *dystrophin* mRNA. **a** *Schematic* of sgRNA targeting and skipping of mouse *Dmd* exon 23 and location of primers for RT-PCR analysis. Skipping of exon 23 will generate in-frame mRNA. **b** sgRNA target sites in *Dmd* exon 23. **c** RT-PCR analysis of C2C12 mouse myoblast cells transduced with lentiviruses that encode Cas9 and sgDmd1, 2, 3, or 4. The expected band sizes are 353 bp and 140 bp. *M* molecular marker. **d** Sequence analysis of the 140-bp cDNA band from sgDmd2-treated cells confirmed splicing of exon 22 to exon 24

The ability to cause a gain-of-function activity by inducing exon skipping or exon excision suggested that CRISPR-meditated editing using a single sgRNA might be a useful way to partially rescue function to a disease gene that requires low-level rescue. CRISPR-mediated homologous DNA repair has been used to correct premature stop codon mutations in the *Dmd* gene in a mouse model of DMD [22] and several groups have used CRISPR to delete *Dmd* exons and partially restore *Dmd* expression [23–26]. We designed four sgRNA/Cas9 lentiviruses that target different sites in exon 23 of the *Dmd* gene (Fig. 4a, b) and transduced mouse C2C12 myoblasts, a cell line widely used as a model for Duchenne muscular dystrophy (DMD) [27]. In C2C12 cells transduced with *Dmd* sgRNAs, we detected an RT-PCR product that corresponds to the normal splice product containing exon 23. Sequencing these RT-PCR products revealed that only *Dmd*-sg2 efficiently edited *Dmd* exon 23, as evidenced by mixed sequence peaks beyond the sgRNA target site (Additional file 1: Figure S9). In cells transduced with *Dmd*-sg2, we also detected an RT-PCR product corresponding to exon 22 spliced to exon 24 (Fig. 4c, d). Thus targeting exon 23 with one sgRNA might be sufficient to induce partial exon skipping and produce an intact dystrophin open reading frame. DMD is a classic example of a disease in which a small amount of functional restoration can provide substantial clinical benefit [28].

## Discussion

Whereas gene inactivation is most often the goal of CRISPR-mediated editing, our findings identify exon skipping as an unintended consequence of genome editing. We also show that exon skipping can result from indels that cause alternative splicing or from larger deletions that remove exons. Novel splice isoforms could encode proteins that retain partial function and should be carefully considered when interpreting phenotypes that result from CRISPR-induced mutations.

The frequency with which CRISPR-induced indels cause exon skipping is difficult to predict. Nevertheless, exon skipping caused by point mutations—including nonsense, missense, and translationally silent mutations—is well documented [29–32] and our results complement a recent study, which showed that CRISPR-mediated editing of the human *FLOT1* gene can cause exon skipping by alternative splicing [18]. Roles for nonsense-mediated decay or cis-acting regulatory elements have been proposed, but mechanisms remain elusive. DNA damage has also been shown to regulate exon skipping [33]. Our data do not resolve whether the DNA damage, the indel, or the premature stop codon induces exon skipping, but they are consistent with the model that some indel mutations disrupt cis-acting sequences that promote splicing [29]. Future studies are needed to determine how CRISPR-induced indels cause alternative splicing and identify rules for predicting when exon skipping will occur.

We detected an unexpectedly high frequency of large deletions induced by CRISPR using a single sgRNA. We and others previously showed that two sgRNAs can generate large genomic deletion or inversion [34, 35]. However, large deletions induced by a single sgRNA have not been systematically analyzed in the literature. We initially missed these large deletions with the short-range PCR assays typically used to genotype CRISPR clones. We therefore recommend that, whenever possible, long-range PCR be used to genotype CRISPR clones. In many cases, large deletions will disrupt gene function and accomplish the goal of a CRISPR-mediated genome editing experiment. But our findings warrant careful analysis of editing events, because the aberrant juxtaposition and splicing of exons could result in neomorphic alleles.

Although exon skipping is an unintended consequence of CRISPR-mediated editing, we have shown that exon skipping can produce mRNAs that encode gain-of-function or partially functional proteins. Thus, exon skipping induced by CRISPR-mediated editing might be harnessed as a way to restore partial function to disease genes, in much the way that exon skipping induced by antisense oligonucleotides is being explored as a therapeutic to treat genetic diseases that result from splicing mutations [36].

## Methods

### CRISPR vectors

sgRNAs (Additional file 1: Table S1) were cloned into the lentiV2 (Addgene 52961) or pX330 (Addgene 42230) vectors using standard protocols [37].

### Cell culture and infection

Cell culture conditions were as described [34]. A total of 293 fs cells were used to package lentiviruses encoding individual sgRNA and Cas9. KP cells or C2C12 cells were infected with lentiV2 lentiviruses and selected with puromycin. For Fig. 3a, cells were transduced with sgCtnnb1.2 cloned into lentiGuide-Puro vector (Addgene 52963), lenti Cas9-Blast (Addgene 52962), dCas9-BFP (Addgene 46910), or dCas9-KRAB-BFP (Addgene 46911). Cells were selected with puromycin, blasticidin, or FACS sorted for BFP.

### Isolation of single-cell clones

KP or NIH-3T3 cells were transduced with lentiviruses Cas9 and sgRNAs targeting *Kras*, *Ctnnb1*, or *p65* and were selected with puromycin for four days. For each transduction, 500 puromycin-resistant cells were seeded into a 100-mm dish and cultured until cell colonies were observed under a microscope. Individual colonies were transferred to 12-well plates—one colony per well—and grown to confluence. Genomic DNA and total RNA was isolated and PCR or RT-PCR was used to identify clones with indels, deletions, or insertions and exon skipping. Genomic PCR products were cloned into a TOPO vector to sequence alleles with indels or deletions.

### CRISPR-induced insertion/deletion detection

Genomic DNA from cells was harvested by quick extraction buffer (Epibio), sgRNA target sites were amplified by PCR, and the products were sequenced on an Illumina NextSeq 500 [34]. We mapped the reads to the reference sequence using BWA (version 0.7.5) and SAMtools (version 0.1.19). VarScan2 (version 2.3) was used to identify insertions and deletions with the “pileup2indel” mode and parameters “--min-var-freq,” “--min-avg-qual,” and “--*p*-value.”

### RNA-seq and bioinformatics analysis

RNA-seq libraries were generated using Illumina TruSeq kit, as described [38]. Paired-end 75-nt sequencing was performed using NextSeq. Reads were trimmed and primer sequences were removed using Trimmomatic (v 0.30). Reads were aligned to the mm10 genome using STAR (version 2.3.0e) with default parameters and uniquely mapping reads were selected. Redundant read pairs were removed using Samtools (version 0.0.19). For each gene annotated in GENCODE M7, the number of reads per gene was calculated using HTSeq. Percent exon 2 inclusion (Percentage Splicing Index, PSI or Ψ) for *Kras* was calculated as: (exon 1-2 + exon 2-3)/(exon 1-2 + exon 2-3 + exon 1-3). For global alternative splicing analysis, alternatively spliced exons were called using MISO 0.5.3 with default settings [39] and filtered with stringent cutoffs (ΔΨ ≥ 0.2, total reads ≥ 10, and Bayes factor ≥ 10).

### Western blot analysis

Protein lysates from cultured cells were prepared in RIPA buffer with proteinase and phosphatase inhibitors. Proteins were separated on 4–12% NuPage Bis-Tris gels (Life Technologies, NP0321), transferred to nitrocellulose membrane, and probed with 1:1000 anti-β-Catenin antibody (BD 610154) or 1:5000 anti-Actin antibody (CST 8457).

### RT-PCR and TOPO cloning

RNA was purified using RNeasy Mini Kit (Qiagen). First strand cDNA was synthesized using Superscript (ABI) and target sequences were amplified using LA-Taq (Clontech) or Herculase II (Agilent). Primers were listed in Additional file 1: Table S2. Gel bands were quantified using the ImageQuant TL software. Exon skipping products were gel purified, re-amplified using the same PCR protocol to increase yield, and cloned into a TOPO vector. TOPO clones were submitted to Genewiz for sequencing. Representative results from two lentiviral infections are shown.

## Additional files

Additional file 1: Supplemental Figures and Tables 1–3. (PDF 4407 kb)

Additional file 2: Supplemental Table 4. (XLSX 32 kb)
