# Supplementary material for: CRISPR/Cas9-mediated genome editing induces exon skipping by alternative splicing or exon deletion
Source: Genome Biol. 2017 Jun 14;18:108. doi: 10.1186/s13059-017-1237-8 (PMC5470253; doi:10.1186/s13059-017-1237-8)
Supplement: Supplementary file 1 — Supplemental Figures and Tables 1–3. (PDF 4407 kb) [file 13059_2017_1237_MOESM1_ESM.pdf]

Fig. S1

a

	Parental	CRISPR clone
KP1	$Kras^{G12D/+}$	-CG<br>-C deletion
KP2	$Kras^{G12D/+}$	-GG deletion

b

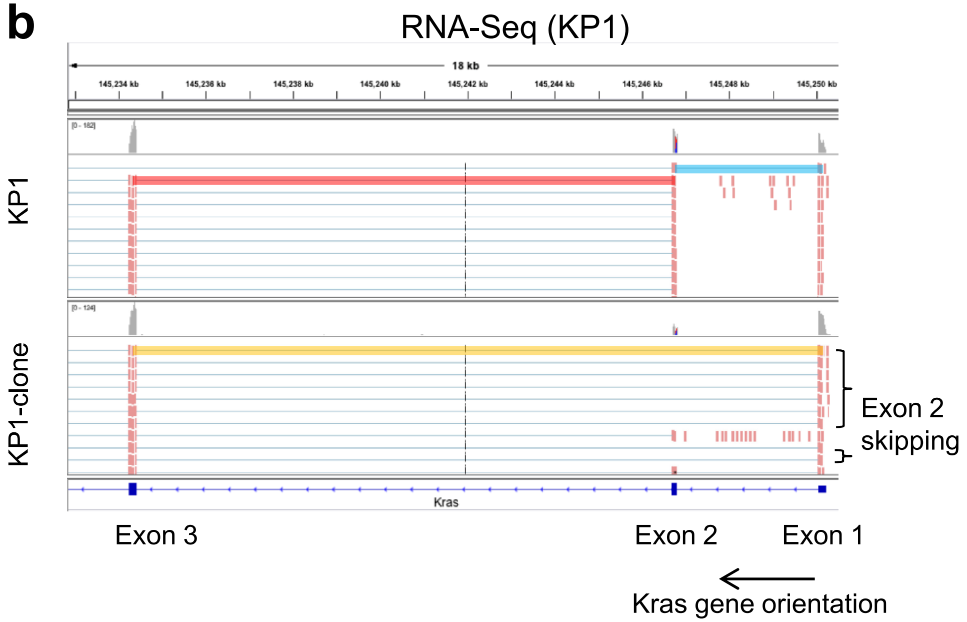

c

d

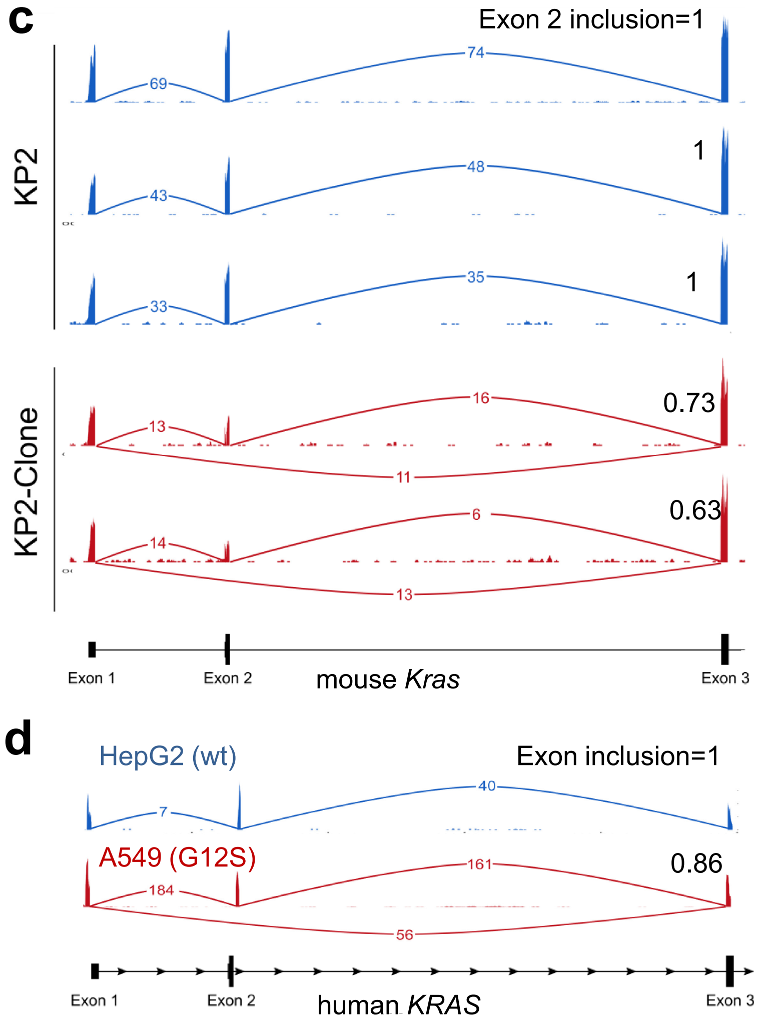

Figure S1. CRISPR-mediated editing of *Kras* induces exon skipping. (**a**) Genotypes of *Kras* CRISPR clones. (**b**) Browser view of representative Kras RNA-Seq results from KP1 parental cells and KP1-clone. Reads that skip exon 2 are indicated. (**c**) RNA-Seq showing partial exon 2 skipping in KP2-clone (n=2), but not in the parental KP2 line (n=3). (**d**) RNA-Seq data showing partial exon 2 skipping of $KRAS^{G12S}$ in the human A549 lung cancer cell line. HepG2 cells show wildtype splicing of *KRAS*.

Fig. S2

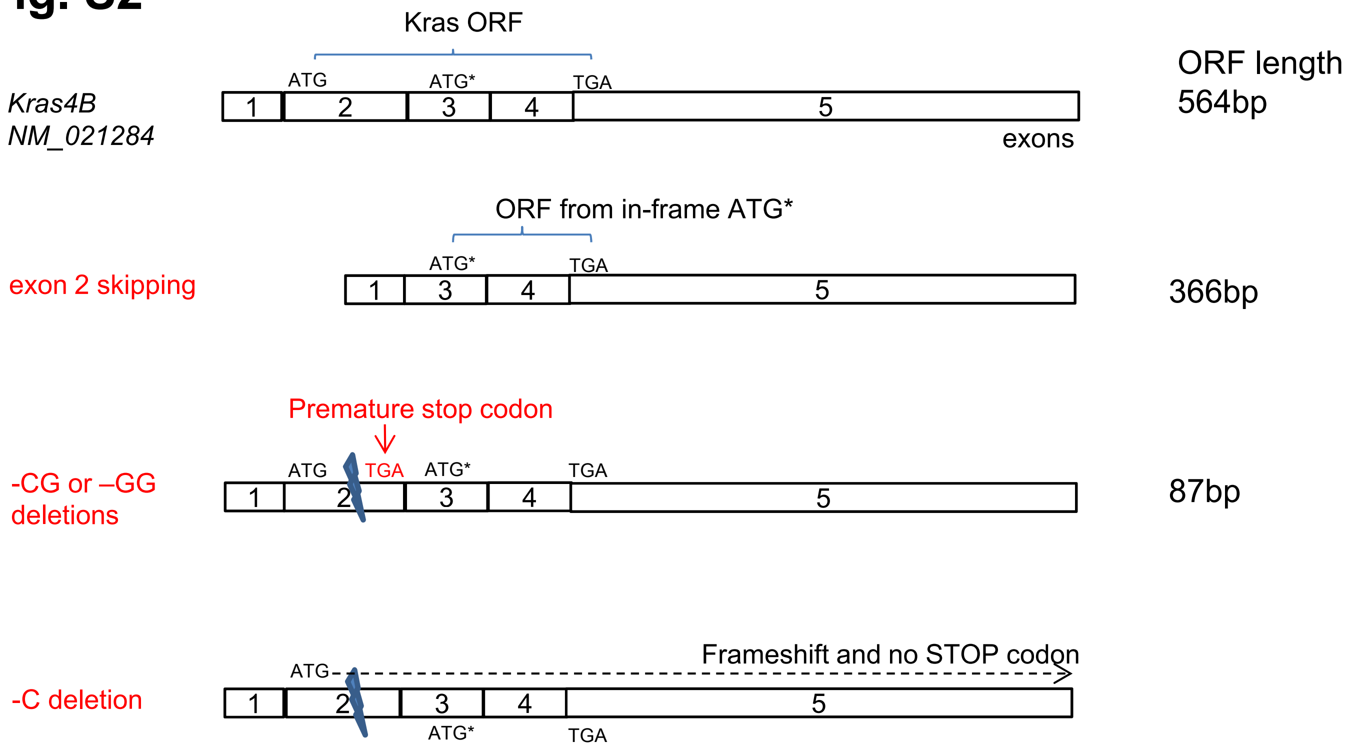

Fig. S2 Reading frame of Kras mRNA with indels or exon 2 skipping. Kras4B is the major isoform of Kras in KP cells. ATG* is an in-frame downstream ATG in exon 3.

Fig. S3

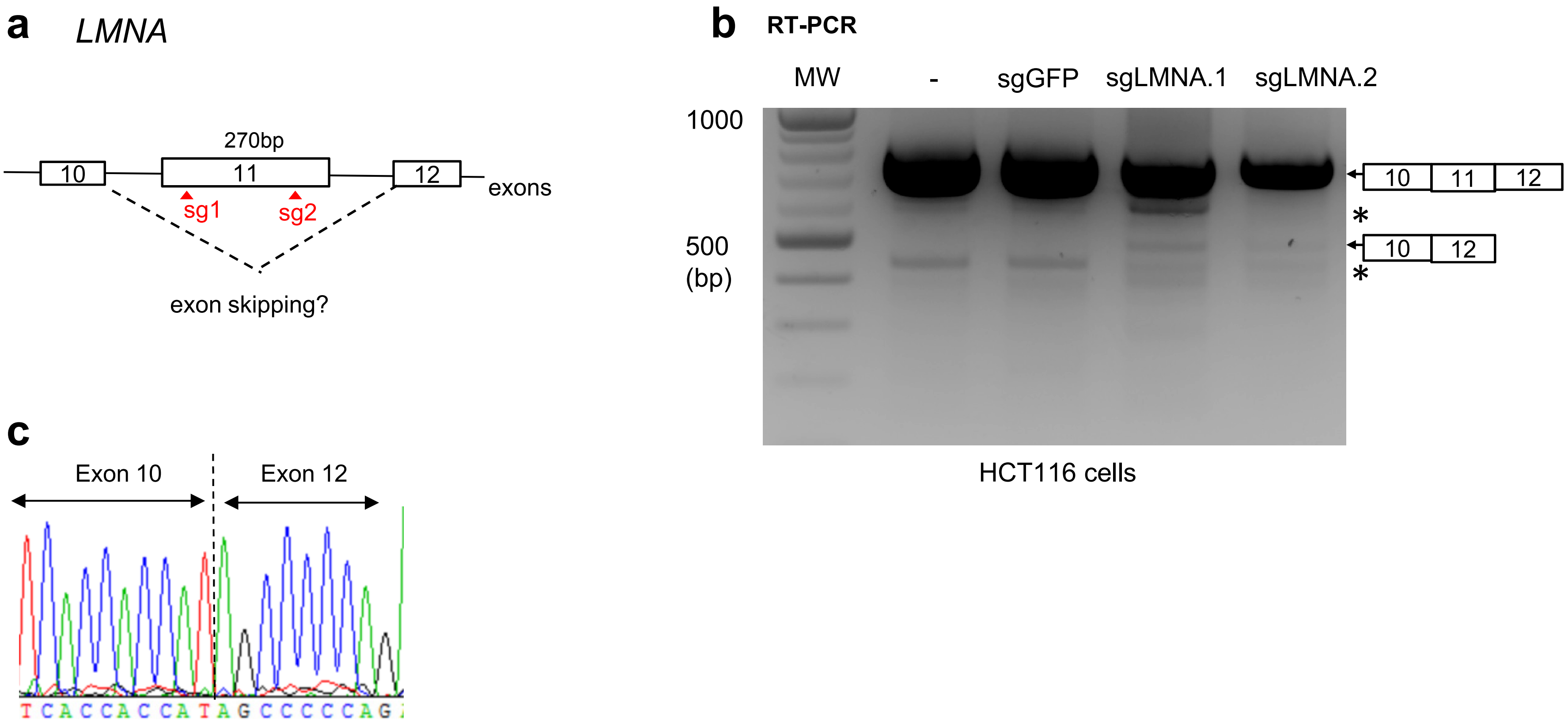

Figure S3. CRISPR induces partial skipping of the *LMNA* exon 11 in human cells. (**a**) Schematic showing the locations of sgRNAs targeting *LMNA* exon 11. (**b**) RT-PCR showing partial skipping of *LMNA* exon 11 in human HCT116 cells transduced with lentivirus encoding Cas9 and sgLMNA.1 or sgLMNA.2. Exon 10-11-12 and exon 10-12 spliced products are indicated. “*” denotes bands of unknown identity. (**c**) Sequence analysis confirmed the identity of the exon 10-12 splice product from (b).

Fig. S4

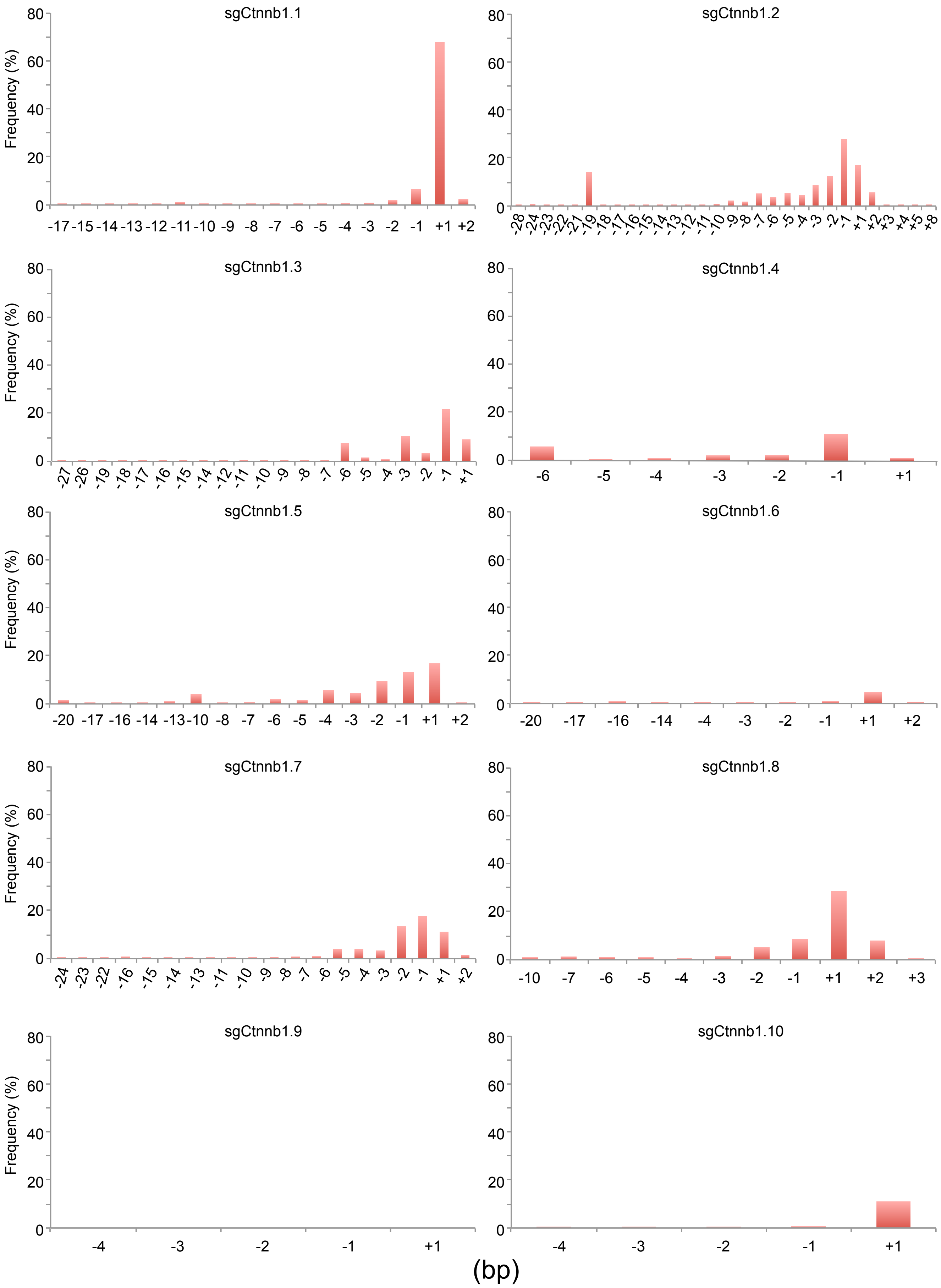

Fig. S4 Length distribution of Ctnnb1 indels. Bar charts showing the frequency of insertion or deletion of the indicated size induced by each Ctnnb1 sgRNA. + denotes insertions; - denotes deletions.

Fig. S5

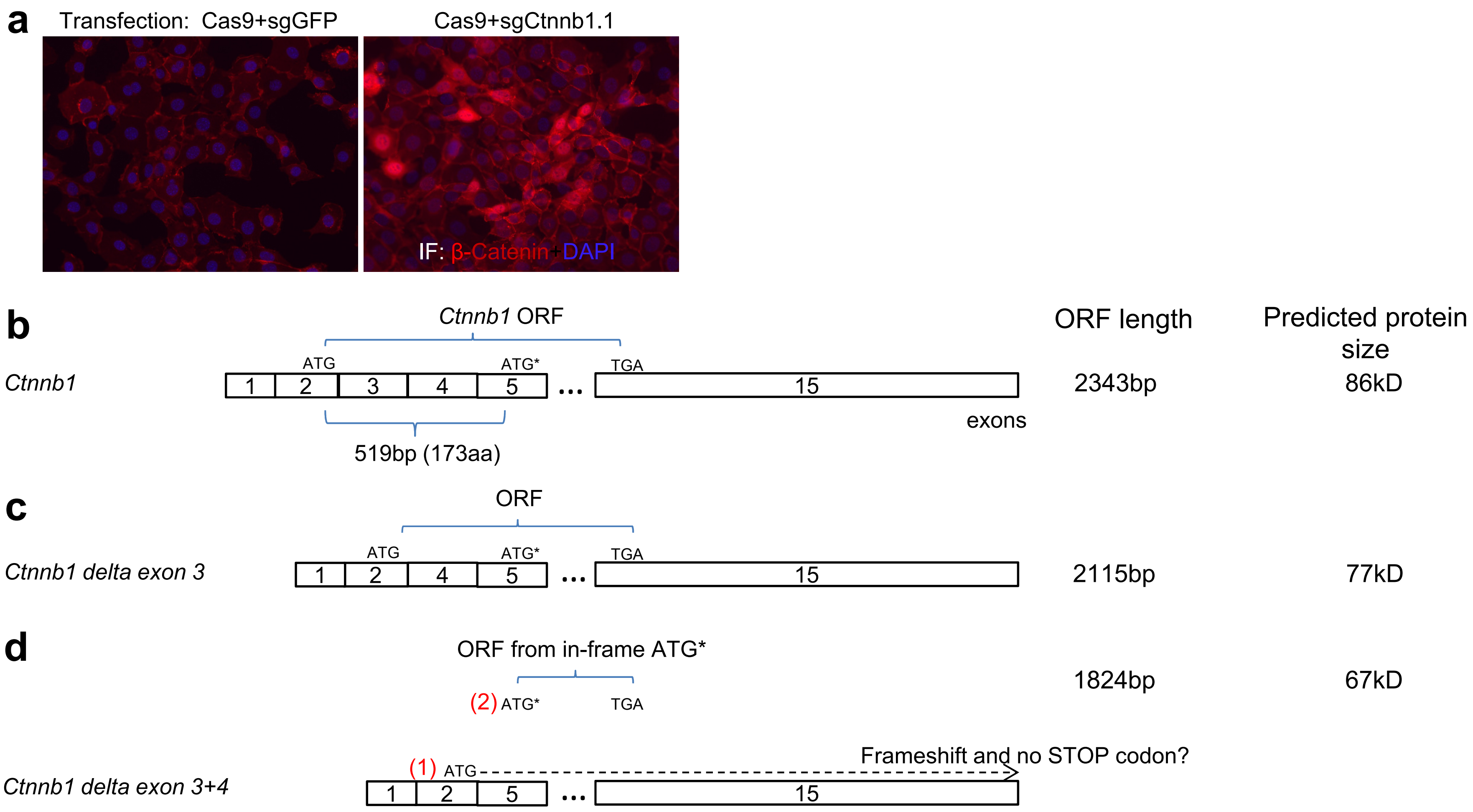

Figure S5. *Ctnnb1* sgRNA induces exon 3 skipping and a constitutively active β-Catenin. (**a**) Immunofluorescence imaging of β-Catenin in mouse fibroblast cells transiently transfected with plasmids expressing Cas9 and sgCtnnb1.1. Cells were fixed on day seven and stained with DAPI and anti-β-Catenin antibody (40× lens). (**b-d**) Ctnnb1 reading frames that result from proper splicing and exon skipping. (**b**) Full length mRNA. (**c**) Skipping exon 3 removes the sequence encoding an auto-inhibitory domain of β-Catenin. (**d**) Skipping of exons 3 and 4 produces a shifted reading frame with no STOP codon (1). The mRNA has a second ORF from an in-frame ATG (2).

Fig.S6

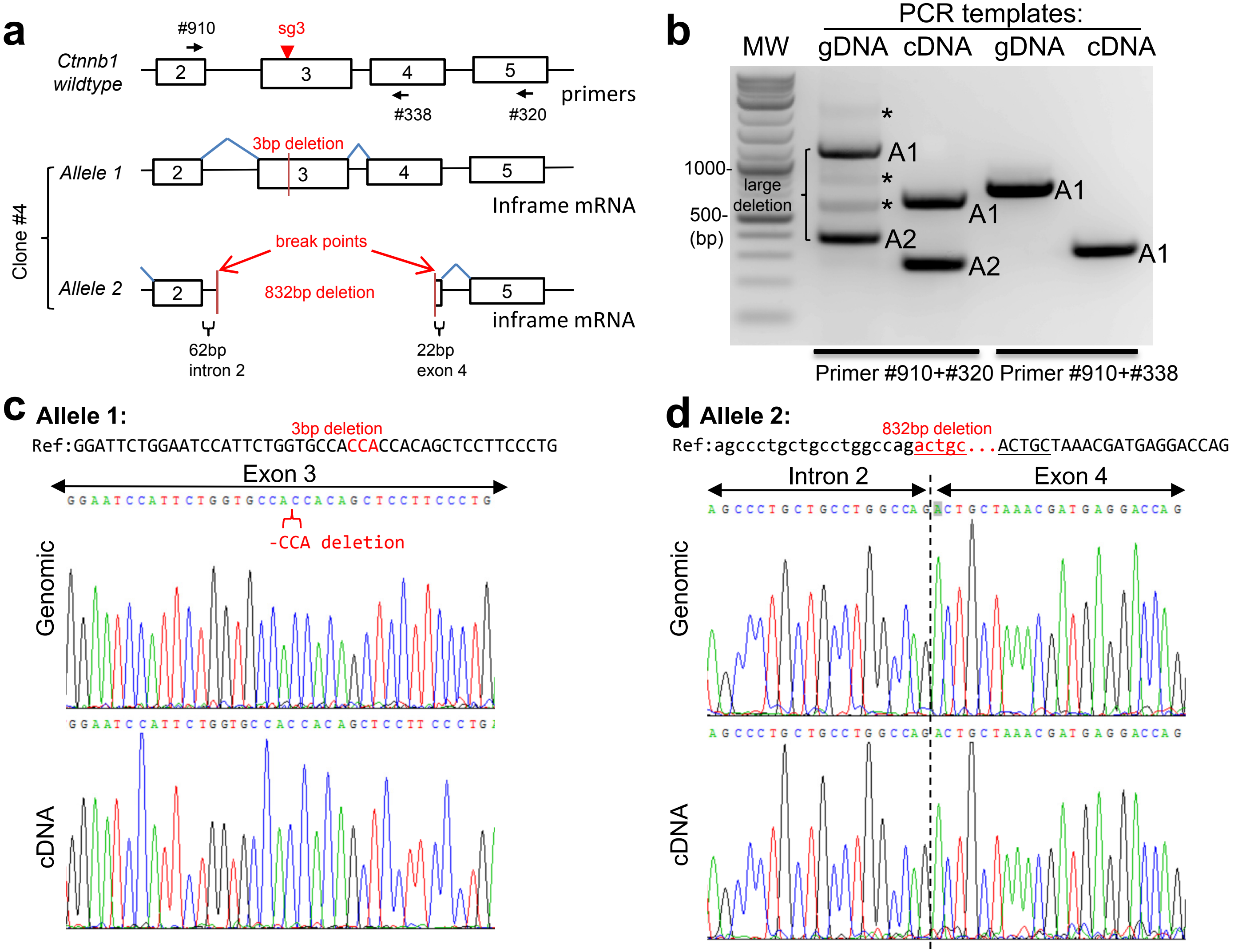

Figure S6. A 3-bp deletion in *Ctnnb1* did not induce exon skipping. (**a**) Diagram of editing events in a cell clone #4: a 3-bp "CCA" deletion in one allele and a 832bp large deletion in the other. (**b**) PCR analysis detects the large deletion. Two sets of primers are shown in (a). Primer #910+#320 detect both alleles (A1 and A2). Primer #910+#338 only detects allele 1 (A1). "*" denotes nonspecific PCR bands. (**c-d**) Genomic and cDNA sequence analyses of Allele 1 (c) and Allele 2 (d). Only two cDNA products were identified: one that corresponds to Allele 1 with the 3-bp deletion, and one that corresponds to the fusion of the intron 2 and exon 4 breakpoints in Allele 2.

Fig.S7

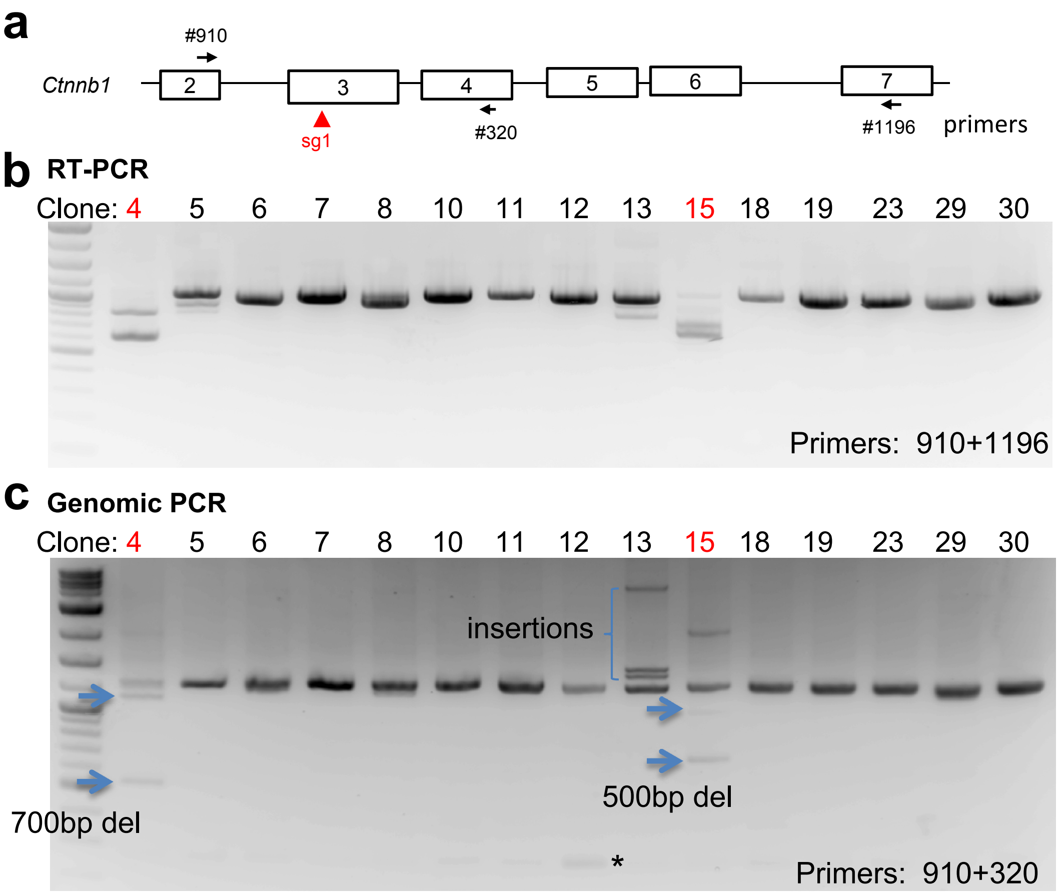

Figure S7. CRISPR with one sgRNA induces larger than expected rearrangements in *Ctnnb1*. (**a**) Diagram of Ctnnb1 gene structure from exons 2 to 7 and positions of the *Ctnnb1*-sg1 target site and primers to analyze *Ctnnb1* cDNA and genomic DNA. (**b**) RT-PCR analysis of 3T3 clones that were isolated after transfection with Cas9 and *Ctnnb1*-sg1. Clones 4, 5, 13, and 15 show smaller cDNA bands. The low levels of full-length RT-PCR bands in clones 4 and 15 might be caused by reduced RNA stability or preferential amplification of smaller RNA bands. (**c**) Genomic PCR of selected clones showed that clones 4 and 15 have genomic deletions (blue arrows). Clone 5 has a 4-bp TCTG deletion. Clones 13 and 15 have insertions. 3T3 cells have a modal chromosome number of 68. "*" denotes nonspecific PCR bands.

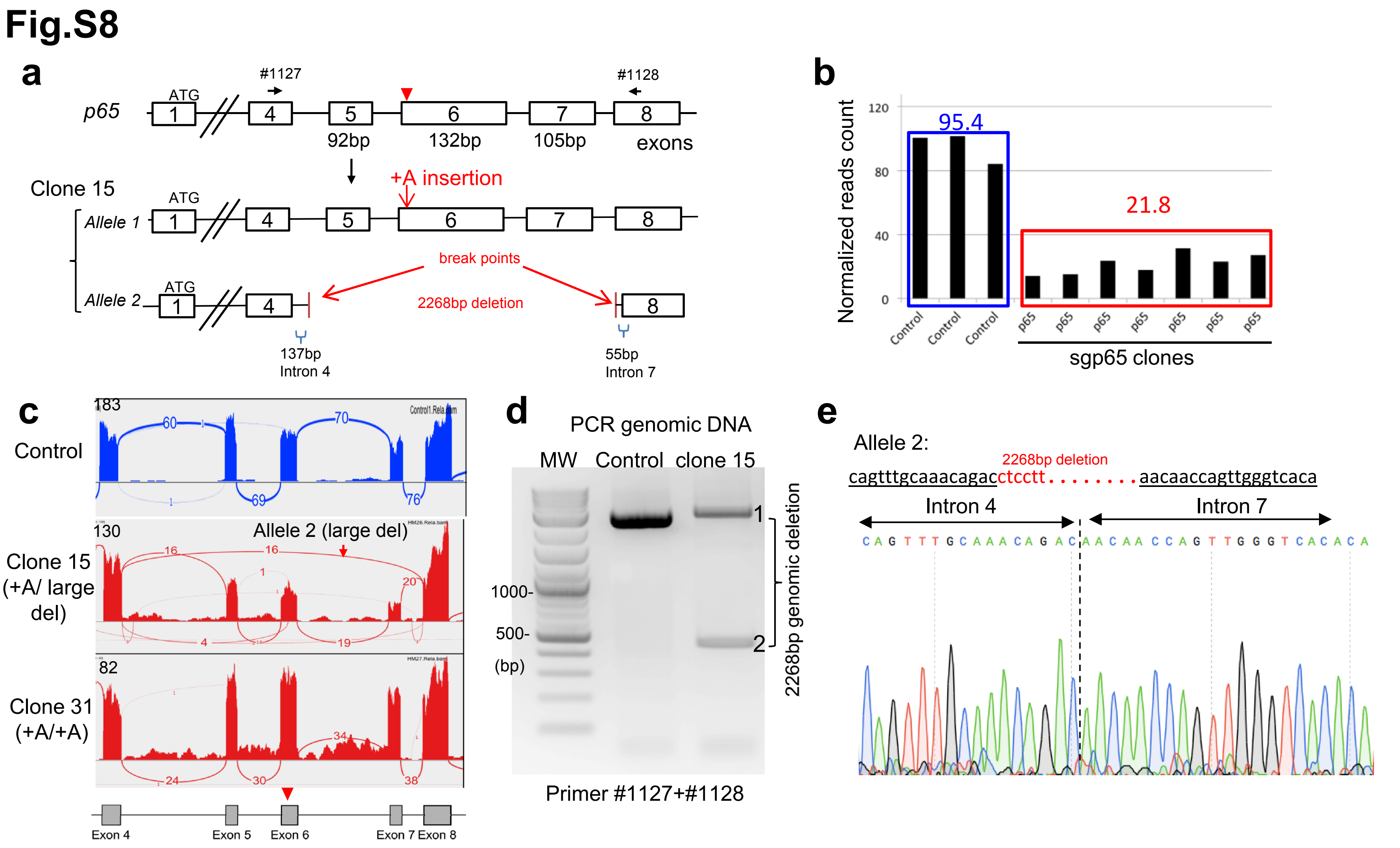

Figure S8. CRISPR with one sgRNA induced a large deletion in *p65*. (**a**) Diagram of p65 exons 1 to 8 and edited alleles in single-cell clone #15: a "+A" insertion in Allele 1, and a 2,268-bp deletion in Allele 2. Positions of the sg*p65* target site (red arrowhead) and primers to analyze *p65* cDNA and genomic DNA are shown. Single-cell clones were isolated from KP cells transduced with lentivirus encoding Cas9 and sg*p65*. (**b**) Bar graph showing that *p65* mRNA reads are reduced in edited *p65* clones compared to control. (**c**) RNA-Seq splicing profiles from control, clone 15, and clone 31 cells. Numbers between exons indicate splice junction reads. Clone 31 has a homozygous A insertion and no exon skipping. (**d**) Genomic PCR detects a shorter band in one allele of clone 15. (**e**) Sequence analysis showing the shorter band is a 2,268bp deletion.

Fig. S9

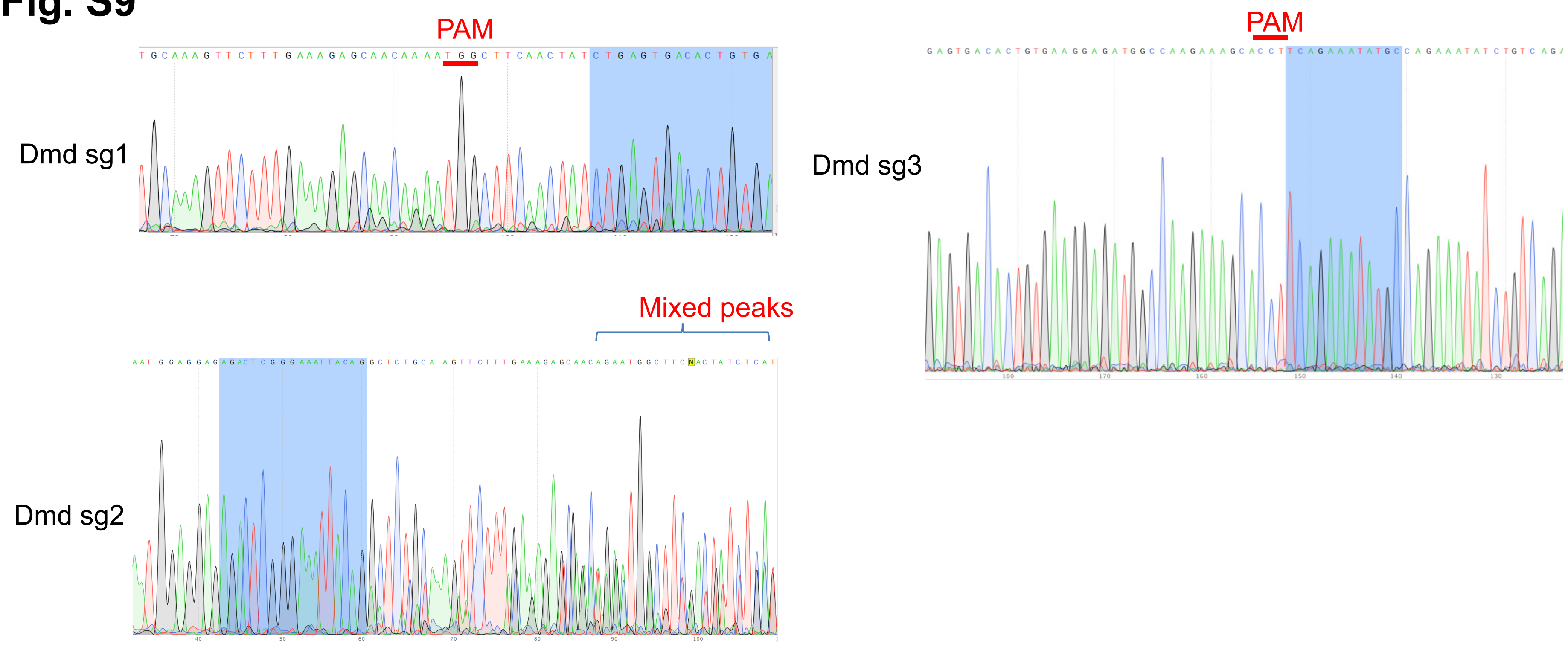

Figure S9. Sequence analysis of the 353-bp *Dmd* RT-PCR bands from Fig. 4C. Mixed peaks in cells transduced with *Dmd*-sg2 indicates the presence of indels. sg1 and sg3 showed clean DNA peaks, indicating that genome editing was inefficient.

Supplemental Information

Supplemental Figure legends:

Figure S1. CRISPR-mediated editing of *Kras* induces exon skipping. (**a**) Genotypes of *Kras* CRISPR clones. (**b**) Browser view of representative Kras RNA-Seq results from KP1 parental cells and KP1-clone. Reads that skip exon 2 are indicated. (**c**) RNA-Seq showing partial exon 2 skipping in KP2-clone (n=2), but not in the parental KP2 line (n=3). (**d**) RNA-Seq data showing partial exon 2 skipping of $KRAS^{G12S}$ in the human A549 lung cancer cell line. HepG2 cells show wildtype splicing of *KRAS*.

Figure S2. *Kras* reading frames that result from indels or exon 2 skipping. *Kras4B* is the major isoform of *Kras* in KP cells. ATG* is an in-frame downstream ATG in exon 3.

Figure S3. CRISPR induces partial skipping of the *LMNA* exon 11 in human cells. (**a**) Schematic showing the locations of sgRNAs targeting *LMNA* exon 11. (**b**) RT-PCR showing partial skipping of *LMNA* exon 11 in human HCT116 cells transduced with lentivirus encoding Cas9 and sgLMNA.1 or sgLMNA.2. Exon 10-11-12 and exon 10-12 spliced products are indicated. "*" denotes bands of unknown identity. (**c**) Sequence analysis confirmed the identity of the exon 10-12 splice product from (b).

Figure S4. Length distributions of Ctnnb1 indels. Bar charts showing the frequency of insertion or deletion of the indicated size induced by each *Ctnnb1* sgRNA.

Figure S5. *Ctnnb1* sgRNA induces exon 3 skipping and a constitutively active β-Catenin. (**a**) Immunofluorescence imaging of β-Catenin in mouse fibroblast cells transiently transfected with plasmids expressing Cas9 and sgCtnnb1.1. Cells were fixed on day seven and stained with DAPI and anti-β-Catenin antibody (40× lens). (**b-d**) Ctnnb1 reading frames that result from proper splicing and exon skipping. (**b**) Full length mRNA. (**c**) Skipping exon 3 removes the sequence encoding an auto-inhibitory domain of β-Catenin. (**d**) Skipping of exons 3 and 4 produces a shifted reading frame with no STOP codon (1). The mRNA has a second ORF from an in-frame ATG (2).

Figure S6. A 3-bp deletion in *Ctnnb1* did not induce exon skipping. (**a**) Diagram of editing events in a cell clone #4: a 3-bp "CCA" deletion in one allele and a 832bp large deletion in the

other. (**b**) PCR analysis detects the large deletion. Two sets of primers are shown in (a). Primer #910+#320 detect both alleles (A1 and A2). Primer #910+#338 only detects allele 1 (A1). "*" denotes nonspecific PCR bands. (**c-d**) Genomic and cDNA sequence analyses of Allele 1 (c) and Allele 2 (d). Only two cDNA products were identified: one that corresponds to Allele 1 with the 3-bp deletion, and one that corresponds to the fusion of the intron 2 and exon 4 breakpoints in Allele 2.

Figure S7. CRISPR with one sgRNA induces larger than expected rearrangements in *Ctnnb1*. (**a**) Diagram of Ctnnb1 gene structure from exons 2 to 7 and positions of the *Ctnnb1*-sg1 target site and primers to analyze *Ctnnb1* cDNA and genomic DNA. (**b**) RT-PCR analysis of 3T3 clones that were isolated after transfection with Cas9 and *Ctnnb1*-sg1. Clones 4, 5, 13, and 15 show smaller cDNA bands. The low levels of full-length RT-PCR bands in clones 4 and 15 might be caused by reduced RNA stability or preferential amplification of smaller RNA bands. (**c**) Genomic PCR of selected clones showed that clones 4 and 15 have genomic deletions (blue arrows). Clone 5 has a 4-bp TCTG deletion. Clones 13 and 15 have insertions. 3T3 cells have a modal chromosome number of 68. "*" denotes nonspecific PCR bands.

Figure S8. CRISPR with one sgRNA induced a large deletion in *p65*. (**a**) Diagram of p65 exons 1 to 8 and edited alleles in single-cell clone #15: a "+A" insertion in Allele 1, and a 2,268-bp deletion in Allele 2. Positions of the sg*p65* target site (red arrowhead) and primers to analyze *p65* cDNA and genomic DNA are shown. Single-cell clones were isolated from KP cells transduced with lentivirus encoding Cas9 and sg*p65*. (**b**) Bar graph showing that *p65* mRNA reads are reduced in edited *p65* clones compared to control. (**c**) RNA-Seq splicing profiles from control, clone 15, and clone 31 cells. Numbers between exons indicate splice junction reads. Clone 31 has a homozygous A insertion and no exon skipping. (**d**) Genomic PCR detects a shorter band in one allele of clone 15. (**e**) Sequence analysis showing the shorter band is a 2,268bp deletion.

Figure S9. Sequence analysis of the 353-bp *Dmd* RT-PCR bands from Fig. 4C. Mixed peaks in cells transduced with *Dmd*-sg2 indicates the presence of indels. sg1 and sg3 showed clean DNA peaks, indicating that genome editing was inefficient.

Supplemental Tables

Table S1 sgRNA sequences.

Table S2 Primer sequences.

Table S3 22 common alternative cassette exon events in the global alternative splicing analysis.

Table S4 Next-generation sequencing data of *Ctnnb1* indels in Figure 2 (excel file).

Table S1. sgRNA sequences. An extra "G" is added for U6 transcription.

sgRNA ID	Sequence (5' to 3')
GFP	gggcgaggagctgttcaccg
Ctnnb1.1	gctgtggtggtggcaccagaa
Ctnnb1.2	gagctccttccctgagtggca
Ctnnb1.3	gcagggaaggagctgtggtgg
Ctnnb1.4	gatggagttggacatggcca
Ctnnb1.5	gaaaagctgctgtcagccac
Ctnnb1.6	gctggcagcagcagtcttact
Ctnnb1.7	gttgcccttgccactcaggga
Ctnnb1.8	gccattcataaaggacttggg
Ctnnb1.9	gtcctttatgaatgggagca
Ctnnb1.10	gcctcccaagtcctttatgaa
Ctnnb1.11	gttcacgcaagagcaagtagc
Kras	gtggttggagctgatggcgt
p65.5	gcagggaaggagctgtggtgg
Dmd.1	gtctttgaaagagcaacaaaa
Dmd.2	gctatctgagtgacactgtga
Dmd.3	gtatttctggcatatttctga
Dmd.4	gcagaatttgaagagattgag
LMNA.E11.1	gagcgcaggttgtactcagcg
LMNA.E11.2	gagttgcccaggaggtaggag

Table S2. Primer sequences.

Primer ID	Sequence (5' to 3')	Notes
910Ctnnb1.F2	cgtggacaatggctactcaag	RT-PCR, Exon 2 forward
Ctnnb1.R5	gaaaggttgtgcagagtccc	Exon 5 reverse

Ctnnb1.R7	ttcagcactctgcttgtggtc	Exon 7 reverse
685Kras.F	tgagacggcaggggaagg	RT-PCR, Exon 1 forward
686Kras.R	tgacctgctgtgtcgagaat	RT-PCR, Exon 3 reverse
921Dmd_Ex22_F	gatccagcagtcagaaagcaaactc	RT-PCR
922Dmd_Ex24_R	tcaggaaaacatcaacttcagcca	
Actin-F	gtgtgacgttgacatccgtaa	RT-PCR
Actin-R	ccaccgatccacacagagta	
LMNA.Exon 7F	CCGTGGAGGAGGTGGATGAG	RT-PCR
LMNA Exon 12R	GCCCCCTCCCATGACGTGCA	

Table S3. 22 common alternative cassette exon events in the global alternative splicing analysis

event_name	ctrl1_psi	sgKras1 _psi	ctrl1_psi-sgKras1_psi	sgKras2_psi	sgKras2 _psi	ctrl2_psi-sgKras2_psi
chr2:102853015:102853236:-(	0.52	0.15	0.37	0.38	0.07	0.31
chr2:102853015:102853236:-(	0.36	0.06	0.3	0.28	0.04	0.24
chr3:37011568:37011794:+@c	0.65	0.31	0.34	0.28	0.03	0.25
chr4:31992387:31992516:+@c	0.71	0.49	0.22	0.63	0.35	0.28
chr12:76099230:76099512:+@	0.42	0.71	-0.29	0.48	0.9	-0.42
chr14:36896255:36896344:-@	0.28	0.73	-0.45	0.5	0.87	-0.37
chr8:45045031:45045168:+@c	0.15	0.36	-0.21	0.21	0.44	-0.23
chr18:65165572:65165646:+@	0.32	0.12	0.2	0.36	0.06	0.3
chr7:128448121:128448208:-(	0.67	0.21	0.46	0.39	0.16	0.23
chr19:47625305:47625484:+@	0.57	0.2	0.37	0.39	0.02	0.37
chr6:145250049:145250231:-(	0.99	0.32	0.67	0.99	0.33	0.66
chr2:102853015:102853236:-(	0.7	0.09	0.61	0.5	0.05	0.45
chr5:138979146:138979272:-(	0.45	0.08	0.37	0.48	0.03	0.45
chr4:131967788:131967975:-(	0.79	0.54	0.25	0.44	0.2	0.24
chr2:180031361:180031528:+	0.63	0.87	-0.24	0.69	0.91	-0.22
chr2:32606961:32607111:+@c	0.75	0.24	0.51	0.38	0.06	0.32
chr2:156514028:156514210:+	0.51	0.72	-0.21	0.4	0.73	-0.33
chr7:128448121:128448208:-(	0.8	0.3	0.5	0.53	0.22	0.31
chr3:27148993:27149072:-@c	0.26	0.47	-0.21	0.25	0.68	-0.43
chr5:23979488:23979647:-@c	0.62	0.9	-0.28	0.62	0.88	-0.26
chr11:120713693:120713767:-	0.19	0.46	-0.27	0.24	0.64	-0.4
chr1:97884126:97884197:-@c	0.31	0.55	-0.24	0.34	0.75	-0.41
